# Oxidative Stress in Aging Brain: Nutritional and Pharmacological Interventions for Neurodegenerative Disorders

**DOI:** 10.1155/2018/3416028

**Published:** 2018-02-11

**Authors:** Anna Maria Giudetti, Michel Salzet, Tommaso Cassano

**Affiliations:** ^1^Department of Biological and Environmental Sciences and Technologies, University of Salento, 73100 Lecce, Italy; ^2^Inserm, U-1192 - Laboratoire Protéomique, Réponse Inflammatoire et Spectrométrie de Masse (PRISM), University of Lille, 59000 Lille, France; ^3^Department of Clinical and Experimental Medicine, University of Foggia, 71100 Foggia, Italy

A large and convincing body of evidence demonstrated that aging is characterized by a progressive decline in the efficiency of physiological functions due to the consequence of free radical-induced damage to cellular macromolecules. Moreover, the age-dependent inability to counterbalance these changes by endogenous antioxidant defenses can further contribute to the oxidative damages.

Many recent concepts of biology or medicine have underlined the importance of nutrition for the maintenance of “physiological” changes during aging or insults stemming from various degenerative diseases. The role of a balanced nutrition to human health is now well documented in different fields such as neuroscience.

Epidemiological analysis of the relations between nutrient consumption and neurodegeneration is complex and it is highly unlikely that a single component might play a major role. In addition, since multiple factors across the human lifespan might influence the brain function in adulthood and in the elderly, multidomain interventions might be more promising in the prevention of the neurodegeneration.

The interactions between lifestyle and brain health have been widely demonstrated, and recent studies have demonstrated the important role of antioxidants and vitamins in the antiaging process and in neurodegenerative disorders, such as Alzheimer's disease (AD) and Parkinson's disease (PD). Moreover, it has been demonstrated that caloric restriction could increase the functional and maximal lifespan, and accumulating evidence demonstrated that the selection of appropriate whole foods or the addition of antioxidants into the diet might be beneficial to increase the functional lifespan, if not the maximal lifespan. One could then argue that caloric selection may be as important as caloric restriction.

Along this line, the current special issue provides appreciable evidence showing that nutritional and pharmacological interventions based on their antioxidant properties might be particularly relevant for the onset and progression of neurodegenerative disorders.

The present volume, at which contributed eminent scholars, includes 10 original research articles and was organized in the attempt to cover the whole field of the interrelationship between oxidative stress, nutritional/pharmacological interventions, and neurodegenerative disorders.

To this regard, an interestingly cross-sectional analysis conducted by L. K. Mischley and colleagues on 1053 individuals affected by idiopathic PD demonstrated that a reduced rate of PD progression is associated to the consumption of different foods, including fresh vegetables, fresh fruit, nuts and seeds, nonfried fish, olive oil, wine, coconut oil, fresh herbs, and spices.

Studies have demonstrated that an altered arginine metabolism is involved in aging and neurodegenerative processes. M. Mazlan and colleagues found a chemical- and region-specific age-related alteration of L-arginine metabolism in the brain of old rats. Moreover, the authors reported that a three-month oral supplementation with vitamin E, in the form of a tocotrienol-rich fraction, had beneficial effects on memory and motor function in old rats. The authors suggested that such effect was related to a reversion of age-associated changes in arginine metabolites in the entorhinal cortex and cerebellum.

Many efforts and resources have been spent in the last years to find new therapeutic strategies for the treatment of neurodegenerative disorders. Since most of these disorders have oxidative and inflammatory components, studies are geared in the finding of strategies to dampen these processes.

It is widely accepted that the main hallmarks of AD are not only senile plaques and neurofibrillary tangles, but also reactive astrogliosis, which contributes to neuronal loss depriving neurons of the homeostatic support. Palmitoylethanolamide (PEA) is an endogenous fatty acid amide, belonging to the class of nuclear factor agonists. PEA has been demonstrated to bind a nuclear receptor and to exert a great variety of biological functions. PEA has been shown to have anti-inflammatory, antinociceptive, neuroprotective, and anticonvulsant properties. M. R. Bronzuoli and colleagues found that PEA is able to dampen reactive astrogliosis and to promote the glial neuro supportive function, thus furnishing an alternative treatment approach for innovative therapeutic strategy against AD.

The study by S. An and colleagues described in a mouse model of AD the neuroprotective effects of mycelium polysaccharides (AMPS) obtained from *Armillaria mellea*, an edible fungus. In particular, AMPS reduced the apoptosis rate, amyloid beta (A*β*) deposition, oxidative damage, and phospho-Tau aggregations in the hippocampus of a murine model of AD. Moreover, AMPS enhanced horizontal movements in an autonomic activity test, improved endurance times in a rotarod test, and decreased escape latency time in a water maze test.

In the study by A. N. Winter and colleagues, the authors demonstrated that a whey protein supplement significantly protects neurons against diverse inducers of oxidative stress, providing the cystine for the synthesis of the endogenous antioxidant glutathione (GSH). The effect of the supplement was dependent on *de novo* GSH synthesis since the neuroprotection was blocked by the inhibition of *γ*-glutamyl-cysteine ligase, the first enzyme of the cellular eliminate GSH biosynthetic pathway.

Furthermore, D. Vergara and colleagues reported that resveratrol treatment led to a significant increase of GSH level and a reduction of GSSG/GSH ratio, as well as of reduced free thiol content in fibroblasts from parkin-mutant early-onset PD patients.

P. Priore and colleagues reported that oleic acid (OA) and hydroxytyrosol (HTyr), two different components of extra virgin olive oil, could affect lipid synthesis in C6 glioma cells. In particular, OA and HTyr inhibited both de novo fatty acid and cholesterol synthesis without affecting cell viability. Interestingly, the effect on lipid synthesis was more pronounced when OA and HTyr were coadministered to cells, suggesting a synergistic effect.

In the study by Z. Hou and colleagues, an edible bird's nest (EBN), traditionally used for general well-being, was used to improve the cognitive decline in ovariectomized rats compared to estrogen-treated rats. The results indicated that 12-week treatment with EBN was able to improve learning and memory in ovariectomized female rats without inducing liver toxicity, a side effect observed in the estrogen-treated group. The latter results suggest that EBN might be an effective alternative to estrogen therapy for menopause-induced aging-related memory loss.

It should be considered that the chronic use of drugs, approved to alleviate the symptoms of several neurodegenerative diseases, is often associated with debilitating side effects. Moreover, none of these drugs seems to stop the progression of the degenerative process. In keeping with this, the finding of new natural molecules useful for the treatment/preventions of neurodegenerative disorders acquires considerable significance. From this special issue, it appears evident that many studies are devoted to the finding of natural molecules directed at improving the symptoms of neurodegenerative diseases. In conclusion, the articles in this special issue offer tantalizing hints at the potential for new prevention strategies and treatments for people with neurodegenerative diseases, encompassing a wide variety of techniques and methods. We hope that these studies will inspire new and useful ideas to fill the gaps that remain in this critical area.

*Anna Maria Giudetti* *Michel Salzet* *Tommaso Cassano*

